# Selective Separation of Acetic and Hexanoic Acids across Polymer Inclusion Membrane with Ionic Liquids as Carrier

**DOI:** 10.3390/ijms20163915

**Published:** 2019-08-12

**Authors:** Bao-Ying Wang, Na Zhang, Zhen-Yu Li, Qiao-Lin Lang, Bing-Hua Yan, Yang Liu, Yang Zhang

**Affiliations:** 1 Waste Valorization and Water Reuse Group, Qingdao Institute of Bioenergy and Bioprocess Technology, Chinese Academy of Sciences, 189 Songling Road, Laoshan District, Qingdao 266101, China; 2 College of Environment and Safety Engineering, Qingdao University of Science and Technology, 53 Zhengzhou Road, Qingdao 266042, China; 3 College of Environmental Science and Engineering, Ocean University of China, 238 Songling Road, Laoshan District, Qingdao 266100, China; 4 University of Chinese Academy of Sciences, Beijing 100049, China

**Keywords:** polymer inclusion membrane, ionic liquids, volatile fatty acids (VFAs), acetic acids, hexanoic acids

## Abstract

This paper first reports on the selective separation of volatile fatty acids (VFAs) (acetic and hexanoic acids) using polymer inclusion membranes (PIMs) containing quaternary ammonium and phosphonium ionic liquids (ILs) as the carrier. The affecting parameters such as IL content, VFA concentration, and the initial pH of the feed solution as well as the type and concentration of the stripping solution were investigated. PIMs performed a much higher selective separation performance toward hexanoic acid. The optimal PIM composed of 60 wt% quaternary ammonium IL with the permeability coefficients for acetic and hexanoic acid of 0.72 and 4.38 µm s^−1^, respectively, was determined. The purity of hexanoic acid obtained in the stripping solution increased with an increase in the VFA concentration of the feed solution and decreasing HCl concentration of the stripping solution. The use of Na_2_CO_3_ as the stripping solution and the involvement of the electrodialysis process could dramatically enhance the transport efficiency of both VFAs, but the separation efficiency decreased sharply. Furthermore, a coordinating mechanism containing hydrogen bonding and ion exchange for VFA transport was demonstrated. The highest purity of hexanoic acid (89.3%) in the stripping solution demonstrated that this PIM technology has good prospects for the separation and recovery of VFAs from aqueous solutions.

## 1. Introduction

The conversion of organic residual waste into platform chemicals though anaerobic microbial fermentation is considered to be a promising alternative route to replace the petroleum based production of chemicals [1]. Anaerobic microbial fermentation can produce volatile fatty acids (VFAs), which are short chain monocarboxylic acids consisting of six or fewer carbon atoms (e.g., acetic, propionic, butyric, valeric, and caproic (hexanoic) acids) [2]. These VFAs have a wide range of applications such as bioplastic production [3], bioenergy [4] as well as the biological removal of nutrients from wastewater [5]. However, the commercialization of VFA value-added chemicals via fermentation is challenging due to the relatively low VFA concentration in the fermentation broths and the complex fermentation composition [6]. Therefore, the separation and purification of these organic acids from fermentation broths have recently received considerable attention [7].

Different methods have been investigated like salt precipitation [8], solvent extraction [9], liquid membranes (LMs) [10], adsorption [11], microfiltration and/or nanofiltration [12], crystallization [13], and electrodialysis [14], etc. Torri et al. [15] introduced lipophilic amines based LMs for selective conversion of VFAs (acetic, propionic, and butyric acids) from anaerobic fermentation systems and proposed that these LMs had a higher affinity for longer carbon chain VFAs. Similar results were also discovered by Nuchnoi et al. [16], who used a supported liquid membrane (SLM) with tri-n-octyl phosphine oxide (TOPO) as a carrier to separate formic, acetic, propionic, and butyric acids. The results obtained exhibited an obvious difference in transport flux for four VFAs and butyric acid was the easiest to transport across the membrane, followed by propionic, acetic, and formic acids. These studies all demonstrated the feasibility of LMs for VFA selective separation. However, these LMs tended to lose the solvent to the water phases and their lack of stability may hinder their applications [17].

Polymer inclusion membrane (PIM) technology has drawn the considerable attention of many researchers in recent years in the separation of small organic compounds and metal ions from aqueous solutions [18,19,20]. PIMs are a novel type of polymer based liquid membrane where the carrier and plasticizer are incorporated into the entangled chains of the base polymer [21]. The base polymer plays a vital role in providing mechanical strength to the membranes and the carrier is responsible for binding with the interest species and transporting them across the membrane [22]. The plasticizer improves the elasticity, flexibility, and compatibility of the membrane components [23]. It should be pointed out that the majority of carriers in PIMs have plasticizing properties and there is no need to add additional plasticizer to the membrane composition [22,23,24]. The popularity of PIMs is mainly due to their excellent stability than that of other kinds of LMs such as bulk liquids (BLMs), emulsion liquids (ELMs), and supported liquid membranes (SLMs) [25]. Furthermore, PIMs can provide many other advantages in studies such as high selectivity, simple preparation, long term use, excellent stability and versatility, quick transport, and flexible design [23,26,27], and thus possess much potential.

In recent years, ionic liquids (ILs) have attracted much attention as a kind of environmentally friendly solvent. ILs are salts consisting of an organic cation and inorganic or organic anion with a low melting point [28]. ILs have exhibited many unique properties such as selectivity for specific ions, excellent ionic conductivity, non-flammability, electrochemical stability, high thermal stability, and negligible vapor pressure as well as extractability for different organic and inorganic compounds [29], and are therefore favored by many researchers. In addition, ILs as a carrier can also be employed as effective plasticizers [30].

ILs have been reported as a carrier or extractant in solvent extraction and LMs for the separation of VFAs and have shown a superior performance to conventional solvents in terms of extraction and transport efficiency [31,32,33]. Yang et al. [34], using Aliquat 336 (methyltri-n-octylammonium chloride) as an extractant to extract lactic, acetic, propionic and butyric acids, found that Aliquat 336 had more potential to extract VFAs than that of tri-n-octylamine (TOA) as an extractant. In fact, these solvents were found to possess the superiority of high selectivity for VFAs, suitable affinity strength for VFAs, and high biocompatibility toward microbial systems [15]. Furthermore, Aliquat 336 and phosphonium-based ionic liquids like trihexyltetradecylphosphonium chloride (Cyphos IL 101) have recently attracted considerable attention in PIM research for the transport of metal ions and small molecular species [35,36,37,38].

To the best of our knowledge, most of the literature on the use of PIM have focused on the transport of individual VFAs such as lactic acid [24,39], citric acid [40], succinic acid [41], humic acid [42], or of total VFAs (oxalic, tartaric, and lactic acids) [43] from feed solutions. However, the separation of different kinds of VFAs using PIMs has not been reported. Furthermore, although PIMs have many of the advantages as described above, its relatively lower initial flux values or permeability has always been a major challenge [44]. Therefore, attempts have been made by researchers to improve the properties of PIMs such as introducing crosslinking between components [45], applying novel carriers [46], employing nanoscale additives [47], and applying electric fields on both sides of the membrane [48]. Among them, the application of an electric field by combining the PIM and electrodialysis (ED) process seems to be a favorable and effective method [49].

In this study, the investigation of PIMs for VFA (acetic and hexanoic acids as examples) separation was performed. Two hydrophobic ionic liquids, Aliquat 336 and Cyphos IL101, were selected as carriers to synthesis PIMs. Cellulose triacetate (CTA) was selected as the base polymer due to its good mechanical properties and compatibility. The main parameters influencing the separation process such as the effect of composition (carrier type and content), the effect of feed components (pH and acetic and hexanoic acid concentration) as well as the stripping solution components (stripping solution type and concentration) were investigated. In addition, an integrated system combining electrodialysis with PIM to separate both acids were further explored to verify the performance and feasibility of the PIM in VFA separation during electrodialysis. It is believed that this work may offer a method for green and sustainable VFA separation processes.

## 2. Results and Discussion

### 2.1. Transport Mechanism

Acetic acid (pKa = 4.74) and hexanoic acid (pKa = 4.83) exist in two forms (i.e., dissociated and undissociated forms) in aqueous solutions, depending on the solution pH. When the pH < pKa, both acids are protonated and thus exist in undissociated forms. In contrast, dissociated forms are dominant when the pH > pKa [50]. Amine extractants such as TOA and tridodecylamine (TDDA) are typical carriers for carboxylic acids in liquid–liquid extraction and liquid membrane systems [51,52], and only protonated (undissociated) carboxylic acids can be extracted by these extractants through the hydrogen bonding mechanism [9,53]. Nevertheless, the quaternary ammonium Aliquat 336 can extract most of the dissociated and partially undissociated forms of acids because Aliquat 336 is composed of an organic cation associated with a chloride ion [34]. Therefore, coordinating mechanisms are coexist when using Aliquat 336 as the carrier. In conditions of an initial pH 6, the values of pH in the feed solution decreased gradually due to the reverse transport of HCl [54]; when HCl was used as the stripping solution, dissociated (pH > pKa) and undissociated (pH < pKa) forms were observed throughout the operation time. However, the pH of the feed solutions all measured above 6.0 during the experiment when Na_2_CO_3_ was used as the stripping solution. This means that the anion-exchange mechanism was dominant under this condition. In conditions of pH < pKa, the undissociated acids extracted by Aliquat 336 through the interfacial hydrogen bonding mechanism is known by [51]:

(1) $$(R_{4}N^{+}{\mathrm{Cl}}^{-}){}_{\left(\mathrm{mem}\right)}\;+\;\mathrm{HA}{}_{\left(\mathrm{aq}\right)}\;↔\;(R_{4}N^{+}{\mathrm{Cl}}^{-}){\mathrm{HA}}_{\left(\mathrm{mem}\right)}$$

where R_4_N^+^Cl^−^ and HA represent Aliquat 336 and the acetic and hexanoic acid molecules, respectively.

In conditions of pH > pKa, the extraction reaction for the dissociated acid anions by anion- exchange mechanism is described as follows:

(2) $$R_{4}N^{+}{\mathrm{Cl}}^{-}{}_{\left(\mathrm{mem}\right)}\;+\;A^{-}{}_{\left(\mathrm{aq}\right)}\;↔\;R_{4}N^{+}A^{-}{}_{\left(\mathrm{mem}\right)}\;+\;{\mathrm{Cl}}^{-}{}_{\left(\mathrm{aq}\right)}$$

The acids were transferred to the feed/membrane interface and interacted with the carrier to form ionic adducts (Reactions (1) and (2)). The transported compounds are transported through the PIM following a Fickian diffusion pattern [54]. Ultimately, the compounds dissociate immediately at the membrane/stripping interface, according to the reverse reaction of Reactions (1) and (2). The IL molecules return according to their concentration gradient. Compared with acetic acid (Kow of −0.31–0.17), the more hydrophobic hexanoic acid (Kow of 1.88–1.91) is, the easier it reacts with the hydrophobic ionic liquid, thus facilitating its transport.

### 2.2. SEM Analysis

**Figure 1:**
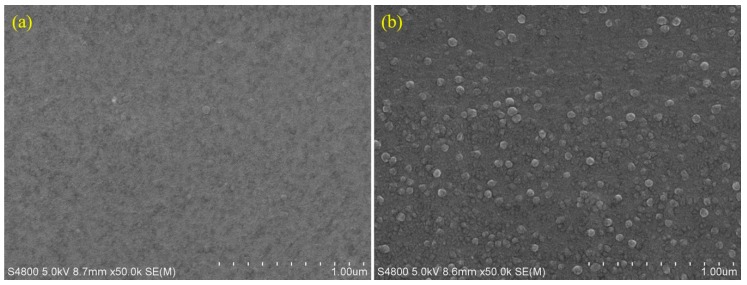
SEM micrographs of the PIMs. (**a**) PIM4; (**b**) PIM6.

Scanning electron microscopy (SEM) images of the prepared PIMs were recorded to observe the surface images of the membrane, as presented in Figure 1. From the SEM images, no pores, holes, or cracks could be observed on the membrane surface, which suggested the homogeneous nature of the prepared membranes. Compared to PIM4, PIM6 exhibited a rougher surface with multiple small particles on the surface, which may be related to the dispersibility of the ionic liquid Cyphos IL 101 in the membrane phase.

### 2.3. Effect of Carrier Type and Transport Kinetics across the PIM

**Figure 2:**
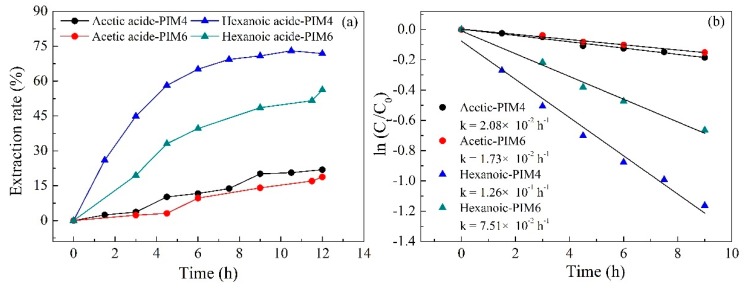
(**a**) Effect of the carrier type (Cyphos IL 101 and Aliquat 336) on the separation of acetic and hexanoic acids from their mixture and (**b**) kinetic plots for the transport of acetic and hexanoic acids across a PIM containing Cyphos IL 101 or Aliquat 336 as the carrier (PIMs: PIM4 and PIM6; Feed solution: 0.01 mol L^−1^ acetic acid + 0.01 mol L^−1^ hexanoic acid, pH = 6; Stripping solution: 0.1 mol L^−1^ HCl).

The nature of the carrier itself is closely related to the properties of the membrane. Therefore, PIMs with two types of ILs (i.e., Aliquat 336 and Cyphos IL 101) as the carrier for the separation of acetic and hexanoic acid were studied in order to investigate the effect of carrier type on the transport of both acids. The extraction rate of the acetic and hexanoic acid is shown in Figure 2a. It can be seen from Figure 2a that the extraction rate of hexanoic acid was much higher than that of acetic acid, regardless of the IL used. As with similar pKa (4.74 and 4.83 for acetic acid and hexanoic acid, respectively), the more hydrophobic the hexanoic acid (octanol–water partition coefficient (Kow) of 1.88–1.91) is, the easier it is to contact the hydrophobic ionic liquid than that of the acetic acid (Kow of −0.31–0.17), and thus easier to transport. This result was also consistent with the results of Torri et al. [15] and Aydin et al. [50], who confirmed that the longer (more lipophilic) the VFA alkyl chain, the higher its transport performance. In addition, the PIM containing Aliquat 336 as the carrier performed a higher extraction rate of hexanoic acid (71.9%) than Cyphos IL 101 as the carrier (56.2%) after 9 h of operation, while the difference in the extraction rate of acetic acid was not obvious. On one hand, this is because the molecular weight of Cyphos IL 101 (519.31) is higher than that of Aliquat 336 (404.17). Thus, the molar amount of ionic liquids in the Aliquat 336 membrane is higher under the same mass. On the other hand, studies have demonstrated that the extraction of carboxylic acids by most ILs in acidic solutions was dominated by hydrogen bonding. The central atom N of Aliquat 336 has an electronegativity of 3.05, which is higher than that of the central atom (P (2.19)) of Cyphos IL 101 [18]. It easily forms hydrogen bonds for Aliquat 336 due to the high electronegativity of N, which decreases the path of acid hopping transport by hydrogen bonding.

The plot of $ln(C_{f}/C_{0})$ versus time for the transport of both acids through the PIM is shown in Figure 2b. Obviously, $ln(C_{f}/C_{0})$ has a linear relationship (R^2^ > 0.98) to time, indicating that the diffusion of acetic and hexanoic acids across the PIM follows the Fick’s first-order kinetics. When the slope is greater, the transport of VFAs from the feed solution to the stripping solution is more rapid. From the slope, the value of the permeability coefficient (P) for hexanoic acids when using Aliquat 336 and Cyphos IL101 as the carrier can be calculated as 4.38 and 2.60 µm s^−1^, respectively (Equation (7)), which were much higher than that of acetic acid (0.72 and 0.60 µm s^−1^, respectively). These results demonstrate the potential of the Aliquat 336 based PIM developed in this study for the selective separation of acetic and hexanoic acids from aqueous solutions.

**Figure 3:**
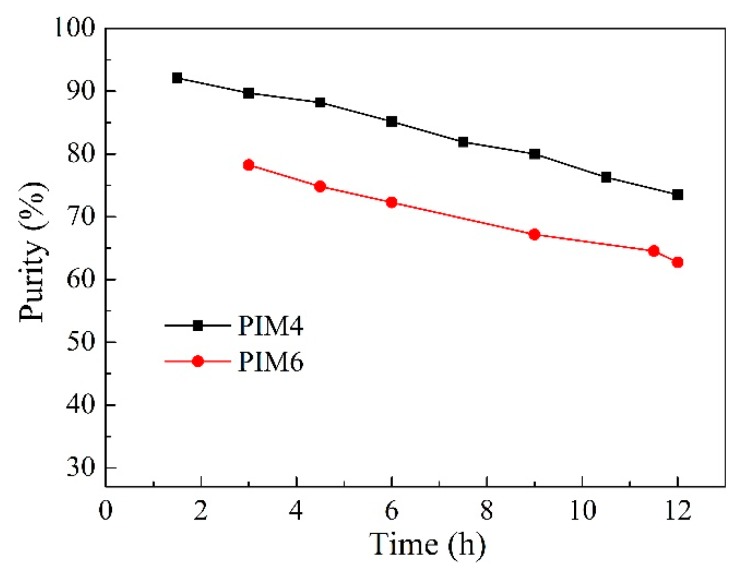
The purity of hexanoic acid in the stripping solution (PIMs: PIM4 and PIM6; Feed solution: 0.01 mol L^−1^ acetic acid +0.01 mol L^−1^ hexanoic acid, pH = 6; Stripping solution: 0.1 mol L^−1^ HCl).

The purity of hexanoic acid in the stripping solution is exhibited in Figure 3. As can be seen from Figure 3, the purity of hexanoic acid when using the Aliquat 336 membrane was much higher than that of the Cyphos IL 101 membrane. The maximum purity of hexanoic acid attained 73.2% and 62.8%, respectively. Considering the excellent performance of the Aliquat 336 membrane during the experiment, IL Aliquat 336 was selected as the carrier for the following experiments.

### 2.4. Effect of Carrier Content

**Figure 4:**
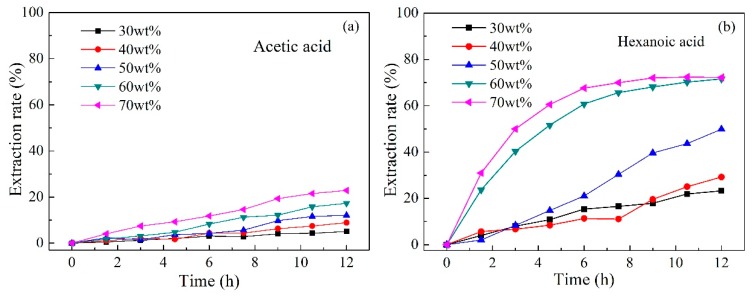
Effect of the Aliquat 336 content on the separation of (**a**) acetic acid and (**b**) hexanoic acid by PIMs (PIMs: PIM1, PIM2, PIM3, PIM4, and PIM5; Feed solution: 0.01 mol L^−1^ acetic acid +0.01 mol L^−1^ hexanoic acid, pH = 6; Stripping solution: 0.1 mol L^−1^ HCl).

To investigate the effect of carrier content on the separation of acetic and hexanoic acids, membranes were prepared with different contents of Aliquat 336 (30, 40, 50, 60, and 70 wt%) while the total mass of the PIMs remained the same. The variation in the extraction rate of acetic and hexanoic acids as a function of Aliquat 336 content is presented in Figure 4. As shown in Figure 4, the extraction rates of acetic acid and hexanoic acid all increased with the increase of Aliquat 336 content in the membrane, which was due to the improvement in the number of available carriers [55]. Since the carrier also has a plasticizing effect in the PIMs, improving its content lowers the diffusive resistance of the membrane, thereby further increasing the extraction rate. In addition, the extraction rate of acetic acid was relatively lower than that of hexanoic acid under the same content of Aliquat 336. Moreover, the difference in extraction rate of hexanoic acid was more pronounced when using varied contents of Aliquat 336. A significant increase in the recovery rate of hexanoic acid from 29.2% to 71.6% was observed as the Aliquat 336 content improved from 40 wt% to 60 wt%, respectively, after 12 h operation. Further increases of carrier content showed no obvious change. This probably indicates that the Aliquat 336 was more evenly distributed in the membrane when the carrier content was 60 wt%, which was previously confirmed by the energy-dispersive X-ray analyzer (EDX) image [18]. Furthermore, the membrane became more viscous for contents higher than 60 wt%, which improved the membrane resistance [56]. 

**Figure 5:**
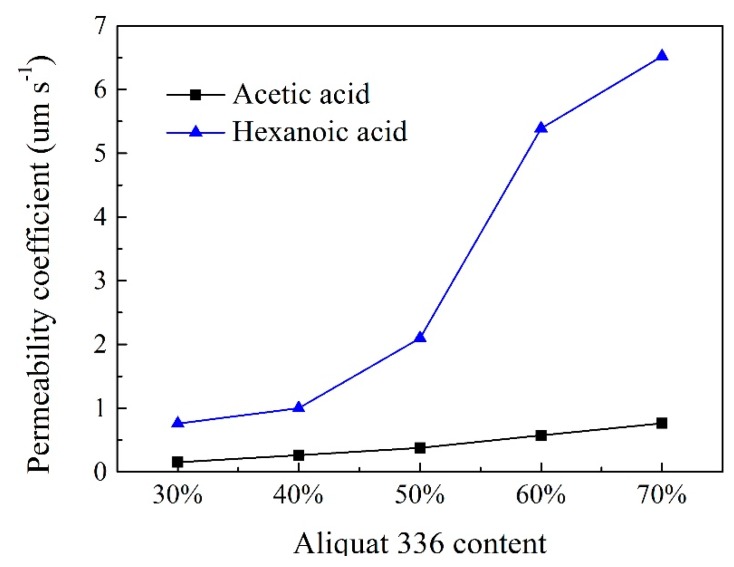
The permeability coefficient of acetic and hexanoic acid by PIMs with different Aliquat 336 content (PIMs: PIMs: PIM1, PIM2, PIM3, PIM4, and PIM5; Feed solution: 0.01 mol L^−1^ acetic acid +0.01 mol L^−1^ hexanoic acid, pH = 6; Stripping solution: 0.1 mol L^−1^ HCl).

Similarly, the permeability coefficient also increased with the carrier content and hexanoic acid showed a much higher value than that of acetic acid (Figure 5). When the content of Aliquat 336 increased from 40 wt% to 60 wt%, the permeability coefficient of hexanoic acid reached 1 µm s^−1^ and 5.39 µm s^−1^, respectively. However, the permeability coefficients of acetic acid were all below 0.77 µm s^−1^, although a higher permeability coefficient could be obtained when the ionic liquid content was 70 wt%. However, judging from the quality of the prepared membrane, the membrane became viscous when the carrier content was higher than 60 wt%. Therefore, a PIM containing 60 wt% Aliquat 366 as the carrier was selected for the subsequent experiments.

### 2.5. Effect of Initial pH in Feed Solution

**Figure 6:**
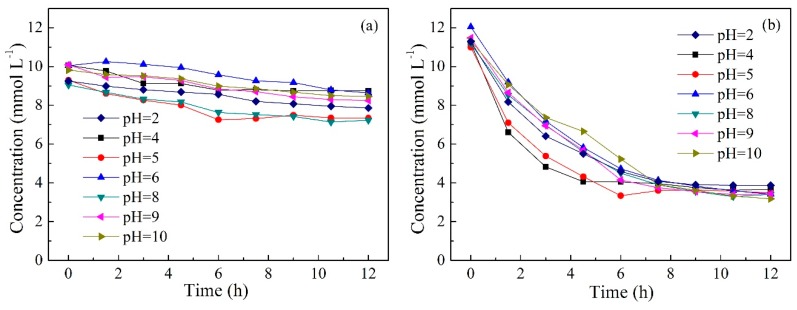
The changes in (**a**) acetic acid and (**b**) hexanoic acid concentrations in the feed solution with different initial pH (PIMs: PIM4; Feed solution: 0.01 mol L^−1^ acetic acid +0.01 mol L^−1^ hexanoic acid; Stripping solution: 0.1 mol L^−1^ HCl).

**Figure 7:**
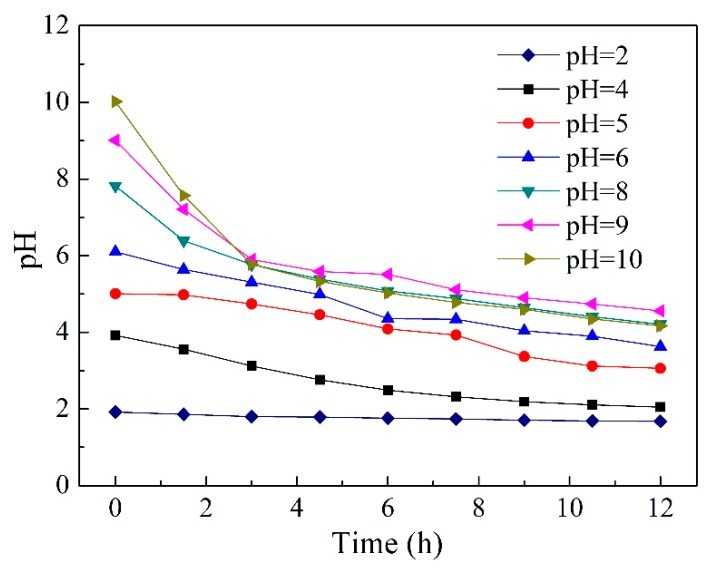
The change of pH in the feed solution at different initial pH values during the experiment (PIMs: PIM4; Feed solution: 0.01 mol L^−1^ acetic acid +0.01 mol L^−1^ hexanoic acid; Stripping solution: 0.1 mol L^−1^ HCl).

As is well-known, pH plays an important role in the extraction and separation of VFAs. Thus, it is essential to understand the effects of pH in the feed solution on the separation of acetic and hexanoic acids though the PIM. pH variation studies in the range of 2.0–10.0 were carried out and the results are illustrated in Figure 6 and Table 1. It can be seen from Figure 6 that the concentration of acetic acid and hexanoic acid in the feed solution decreased over time, regardless of the pH, and as expected, the hexanoic acid concentration decreased more rapidly. For acetic acid, the effect of changes in pH on its transport efficiency was not significant. For hexanoic acid, a relatively lower pH (pH = 4.0–5.0) seemed to be more beneficial to its transport. However, the transport efficiency slowed down under more acidic conditions (pH = 2). This may be due to competitive transport between hexanoic acid and HCl [54]. Studies have demonstrated that most amines extract organic acids from an aqueous solution by forming a hydrogen bond with the undissociated acid [53,57]. Since the concentration of undissociated acid greatly depends on the pH, the organic acid must be set at an acidic condition to be protonated. Nevertheless, a comparison of tertiary and quaternary amines (i.e., TOA and Aliquat 336) for the extraction of VFAs was reported by Yang et al. [34]. The authors discovered that quaternary amine Aliquat 336 could extract both dissociated and undissociated forms of acids, which was different to the tertiary amine TOA. Since Aliquat 336 is comprised of an organic cation associated with a chloride ion, the acid could be extracted by an anion-exchange mechanism when the acid is in a dissociated form (pH > pKa), while the coordinating mechanism by hydrogen bonding is favored when the acid is in an undissociated form (pH < pKa). The change in pH at different initial pH values during the experiment was monitored and the results are illustrated in Figure 7. As shown in Figure 7, the values of pH in the feed solution decreased gradually due to the reverse transport of HCl. In conditions of an initial pH 2.0–4.0, both acids existed in undissociated forms (pH < pKa), and the hydrogen bonding mechanism dominated under these conditions. However, in conditions of initial pH 5.0–10.0, the initial undissociated (pH < pKa) and subsequent dissociated (pH > pKa) forms coexisted throughout the operation time. Thus, acidic acid and hexanoic acid can be transported by both hydrogen bonding and anion-exchange mechanisms under these conditions. Therefore, both acetic and hexanoic acids can be transported though PIMs containing Aliquat 336 as the carrier under the initial pH of acidic and alkali conditions.

The permeability coefficient and purity of acetic and hexanoic acids at different initial pH values are exhibited in Table 1. It can be seen that the permeability coefficient of hexanoic acid was much higher than that of acetic acid. The maximum permeability coefficient of hexanoic acid was shown to have a pH around 4.0, and found to be 6.67 µm s^−1^. Additionally, the purity of hexanoic acid was above 73.5% after 12 h operation. In general, the typical pH range of the fermentation broth in anaerobic acid fermentation varied. Hence, the results demonstrate that the Aliquat 336 based PIM developed in this study may have more potential for the selective separation of VFAs from aqueous solutions.

### 2.6. Effect of Initial Concentration in Feed Solution

**Figure 8:**
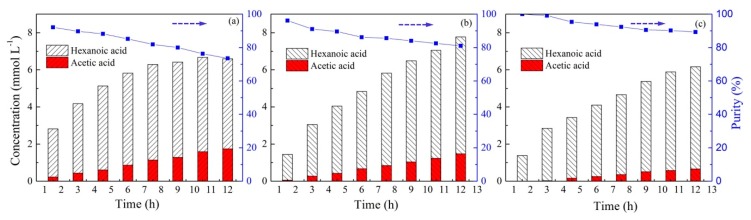
The changes in VFA concentration in the stripping solution with different initial concentrations and the purity of hexanoic acid in the stripping solution (PIMs: PIM4; Feed solution: (**a**) 0.01 mol L^−1^ acetic acid +0.01 mol L^−1^ hexanoic acid; (**b**) 0.03 M acetic acid +0.03 mol L^−1^ hexanoic acid; (**c**): 0.05 mol L^−1^ acetic acid +0.05 mol L^−1^ hexanoic acid, pH = 6; Stripping solution: 0.1 mol L^−1^ HCl).

To evaluate the influence of the initial acid concentration in the feed solution on the selective separation of acetic and hexanoic acids by PIM, experiments were performed by adjusting both acid concentrations from 0.01 mol L^−1^ to 0.05 mol L^−1^. The acid concentration in the stripping solution during 12 h operation is illustrated in Figure 8. As can be seen from Figure 8, an increase in the initial concentration from 0.01 to 0.03 mol L^−1^ was beneficial for the transport of total acids across the membrane, resulting in the highest total acids concentration of 7.77 mmol L^−1^ at a concentration of 0.03 mol L^−1^ (Figure 8b). With a further increase in the initial concentration to 0.05 mol L^−1^, the values of final concentration decreased to 6.16 mmol L^−1^. The reason for the decrease at a higher initial concentration can be explained by the relatively small amount of carrier or the insufficient reaction sites in the membrane, leading to a low extraction rate at a sufficiently high acid concentration [58]. Additionally, as the initial acid concentration increased, the transport of acetic acid decreased. However, it should be noted that the recovery of hexanoic acid remained substantially invariable, which resulting in an improvement in the purity of hexanoic acid in the stripping solution. With an increase in the acid concentration from 0.01 mol L^−1^ to 0.05 mol L^−1^, the purity of hexanoic acid varied from 73.5% to 89.3% after 12 h of operation. The reason for the increase in the purity of hexanoic acid at a higher concentration was due to the intensively competitive transport of both acids. Hexanoic acid with a longer alkyl chain is more hydrophobic, and is therefore more compatible with the hydrophobic IL Aliquat 336 in the membrane [34]. Hence, to achieve a higher acid purity in this system, improving the initial acid concentration may be a favorable choice. 

### 2.7. Effect of Stripping Solution Type

**Figure 9:**
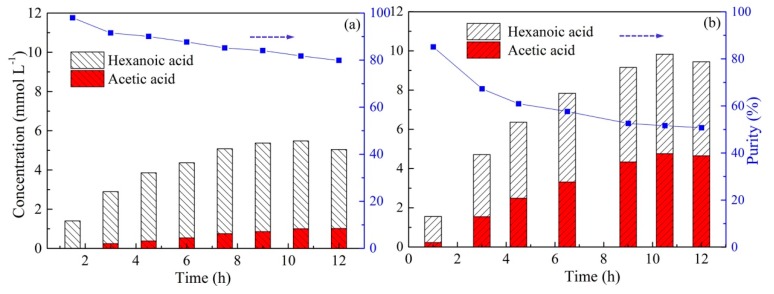
The changes in VFA concentration in the stripping solution with different types of stripping solution (PIMs: PIM4; Feed solution: 0.01 mol L^−1^ acetic acid +0.01 mol L^−1^ hexanoic acid, pH = 6; Stripping solution: (**a**) 0.05 mol L^−1^ HCl; (**b**) 0.05 mol L^−1^ Na_2_CO_3_).

The stripping solution plays a crucial role in the transport of acid ions through PIM because of the decomplexation reaction that occurs at the membrane/stripping interface. Therefore, two different solutions, namely hydrochloric acid and sodium carbonate, with the same concentration were utilized to detect their suitability as a stripping solution. It can be seen from Figure 9 that the difference between using these two types of stripping solution was obvious. Compared to HCl, using Na_2_CO_3_ as the stripping solution achieved a much higher transport efficiency of the total organic acids in the same time. The maximum concentration of using HCl and Na_2_CO_3_ reached 5.04 and 9.44 mmol L^−1^, respectively. When Na_2_CO_3_ was used as the stripping solution, the pH of the feed solution all measured above 6.0 (pH > pKa) during the experiment. This means that the ion-exchange mechanism dominated under this condition. Therefore, a possible reason for the better transport performance may be due to the stronger electronegativity of CO_3_^2-^ than that of chloride ions. In addition, Aliquat 336 is known to transport HCl as an HCl–Aliquat 336 complex [54], which indicates the reverse transport of HCl when utilizing HCl as the stripping solution. Thus, more acetic acid and hexanoic acid radical ions can be transported to the stripping solution when using Na_2_CO_3_ as the stripping solution. In addition, the pH difference between the two aqueous solution provides a large driving force for acid transport in the PIM system [51]. Although the amounts of acetic and hexanoic acids transported all increased when utilizing Na_2_CO_3_ as the stripping solution, the increase of acetic acid was more significant, which resulted in the reduction in hexanoic acid purity (Figure 9b). The optimal purity of hexanoic acid after 12 h of operation for HCl and Na_2_CO_3_ were 81.8% and 50.8%, respectively. These indicate that the selection of an appropriate stripping solution type is crucial for the PIM to achieve more efficient transport and selective separation.

### 2.8. Effect of Acid Concentration in the Stripping Solution

**Figure 10:**
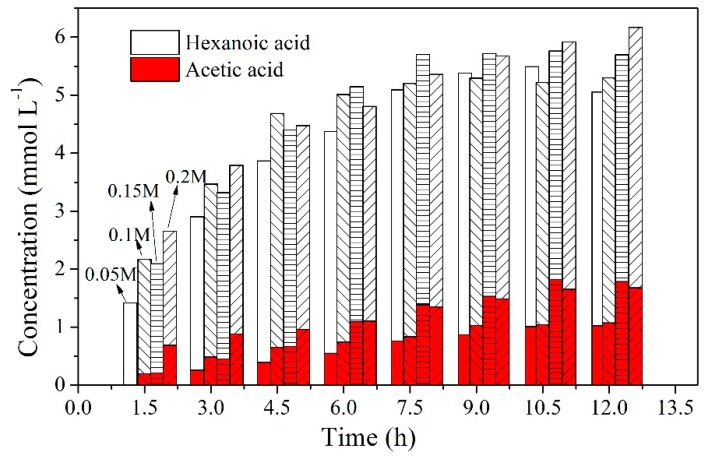
The changes in the acid concentration in the stripping solution with different HCl concentrations in the stripping solution (PIMs: PIM4; Feed solution: 0.01 mol L^−1^ acetic acid +0.01 mol L^−1^ hexanoic acid, pH = 6; Stripping solution: 0.05, 0.1, 0.15 and 0.2 mol L^−1^ HCl).

**Figure 11:**
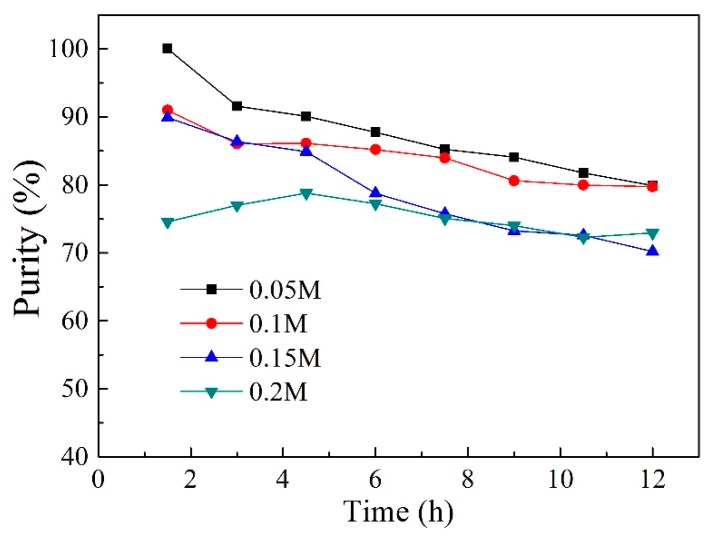
Effect of HCl concentration on the purity of hexanoic acid (PIMs: PIM4; Feed solution: 0.01 mol L^−1^ acetic acid +0.01 mol L^−1^ hexanoic acid, pH = 6; Stripping solution: 0.05, 0.1, 0.15, and 0.2 mol L^−1^ HCl).

The type of stripping solution is also a significant parameter that influences the selective separation. To evaluate the influences of HCl concentration in the stripping solution on the transport of both acids, experiments were carried out by adjusting the HCl concentration from 0.05 to 0.2 mol L^−1^. As shown in Figure 10, variation of the HCl concentration in the stripping solution did not have a dramatic effect on the total transport of both VFAs under these experimental conditions as per the given maximum concentration values, which were 5.03, 5.29, 5.69 and 6.14 mmol L^−1^, respectively, for used HCl concentrations of 0.05, 0.1, 0.15, and 0.2 mol L^−1^. The slight increase was due to the counter transport of protons that provided a driving force for the transport process [55]. However, the effect of HCl concentration on acetic acid was obvious. In particular, with an increase in the HCl concentration from 0.1 to 0.15 mol L^−1^, the concentration of acetic acid in the stripping solution increased dramatically from 1.07 to 1.78 mmol L^−1^, which resulted in the decrease of hexanoic acid purity in the stripping solution. As observed in Figure 11, with an increase in the HCl concentration, the purity values exhibited a decrease tendency. This is possibly related to the large quantitative transport of HCl in the reverse manner, which led to the reduced transport of hexanoic acid in the stripping solution [58]. In general, no discernible benefit was observed by improving the acid concentration, and it is desirable that the acid concentration should be as low as possible for cost and safety reasons. Thus, the optimum acid for back-extraction under transport conditions is 0.05 mol L^−1^HCl.

### 2.9. Adsorption Experiment

**Figure 12:**
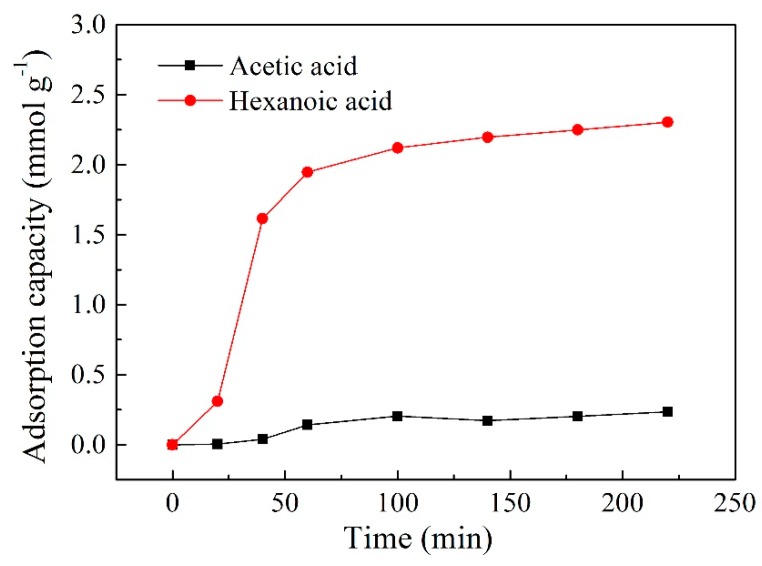
Adsorption capacity of PIM for acetic acid and hexanoic acid (PIMs: PIM4; Feed solution: 0.01 mol L^−1^ acetic acid +0.01 mol L^−1^ hexanoic acid, pH = 6).

To further investigate the transport mechanism of both acids within the PIM using Aliquat 336 as the carrier, an adsorption experiment of the membrane was conducted to verify the difference in transport efficiency between the two acids. As demonstrated in Figure 12, the adsorption amount of hexanoic acid rose rapidly in a short period of time, whereas only a small amount of acetic acid was adsorbed during the same time. The maximum adsorption capacity of hexanoic acid was 2.30 mmol g^−1^, which was 10 times greater than that of acetic acid (2.30 mmol g^−1^). This means that hydrophilic acetic acid finds it difficult to form ionic adducts with the carrier Aliquat 336 in the feed/membrane interface, resulting in a lower transport and recovery efficiency of acetic acid.

### 2.10. Transport Experiment during Electrodialysis

**Figure 13:**
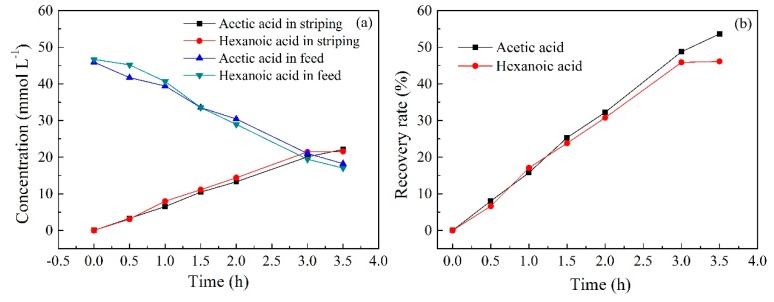
(**a**) The changes of acid concentration in the feed and stripping solution and (**b**) the recovery rate of both acids during electrodialysis (PIMs: PIM4; Feed solution: 0.05 mol L^−1^ acetic acid +0.05 mol L^−1^ hexanoic acid, pH = 6; Stripping solution: 0.1 mol L^−1^ HCl; Current density: 5 mA cm^−2^).

In order to evaluate the feasibility of PIM in the transport and separation of both acids in electrodialysis, a transport experiment combing electrodialysis with the PIM was carried out under a current density of 5 mA cm^−2^. As can be seen in Figure 13a, the transport efficiency was remarkably improved with an electric field was applied when compared with the obtained results without an electric field (Figure 8c). Under the electric field condition, the concentrations of acetic acid and hexanoic acid in the stripping solution reached 22.10 mmol L^−1^ and 21.55 mmol L^−1^, respectively, after 3.5 h of operation. However, the concentration was only 1.38 and 7.22, respectively, after 12 h of operation without the application of an electric field (Figure 8c). This is because the transport of acid ions is accelerated when an electric field is applied. In addition, more acids may be dissociated and quickly transported across the membrane under the effect of an electric field. Nevertheless, according to the presented results, the concentration changes and recovery rates of both acids were very similar to each other (Figure 13a,b). This indicates that the application of an electric field was not beneficial to the selective separation of both acids. Moreover, the application of an electric field may overcome the binding resistance of more hydrophilic acetic acid to the carrier on the membrane, thus greatly increasing the transport efficiency. According to the investigation above, it can be concluded that applying PIM to electrodialysis can dramatically improve the efficiency of the transport of both acids, but is not conducive to their selective separation.

## 3. Materials and Methods

### 3.1. Materials

Cyphos IL 101 (Merck Life Science Co., Ltd, Shanghai, China), Aliquat 336 (Alfa Aesar Chemical Co., Ltd, Beijing, China), CTA (cellulose triacetate) (Acros Organics, New Jersey, USA), and DCM (dichloromethane) (Sinopharm Chemical Reagent Co., Ltd, Shanghai, China) were used to cast the PIMs. Hexanoic acid was purchased from Merck Life Science Co., Ltd. (Shanghai, China). Acetic acid, HCl, H_2_SO_4_, NaOH, and Na_2_CO_3_ used in the experiments were of an analytical grade and purchased from Sinopharm Chemical Reagent Co., Ltd. (Shanghai, China). All chemicals were used without further purification. Deionized water was used to prepare all aqueous solutions. The commercial cation exchange membrane (CJMC-5) was obtained from Hefei Chemjoy Polymer Material Co. Ltd. (Hefei, China).

### 3.2. Membrane Preparation

**Figure 14:**
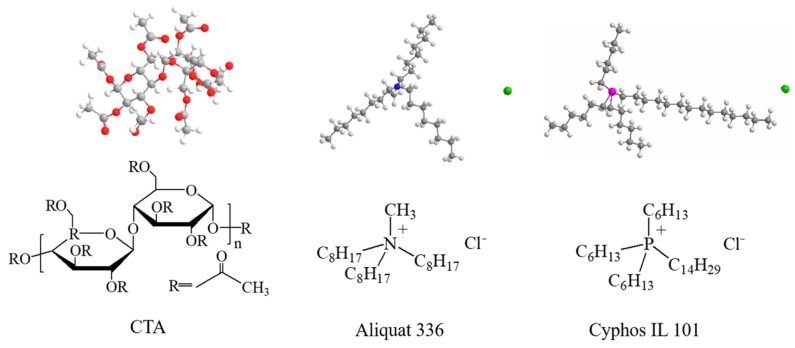
The chemical structures of the polymer and ionic liquids.

PIMs with CTA as the base polymer, and Cyphos IL 101 or Aliquat 336 as the carrier were prepared using the solvent evaporation casting method, and the different compositions and proportions of PIMs studied are listed in Table 2. The composition of each component was varied while ensuring the same total amount of membrane matrix to ensure a consistent membrane thickness. The PIM components with a total mass of 1.1 g was dissolved in 20 mL of DCM solvent, followed by stirring for 2 h using a magnetic stirring bar at room temperature. The uniform casting solution was obtained after degassing for 15 min. The solutions were then poured onto flat glass (13 × 13 cm) placed horizontally, and covered with a watch glass to allow the slow evaporation of the DCM over 12 h. After the evaporation of DCM, the resulting PIMs were then carefully peeled off the glass plate and stored at low temperature for further experiments. The membrane thickness was determined by cutting the membrane in half diagonally and measuring the thickness of 20 points along the cut edge. To acquire knowledge on the surface morphology and characteristics of the membrane, the surface morphology of PIM4 and PIM6 were characterized using scanning electron microscopy (SEM) (Hitachi S-4800, Japan). The chemical structures of CTA and ionic liquids used in the experiment are shown in Figure 14.

### 3.3. Transport Experiments

**Figure 15:**
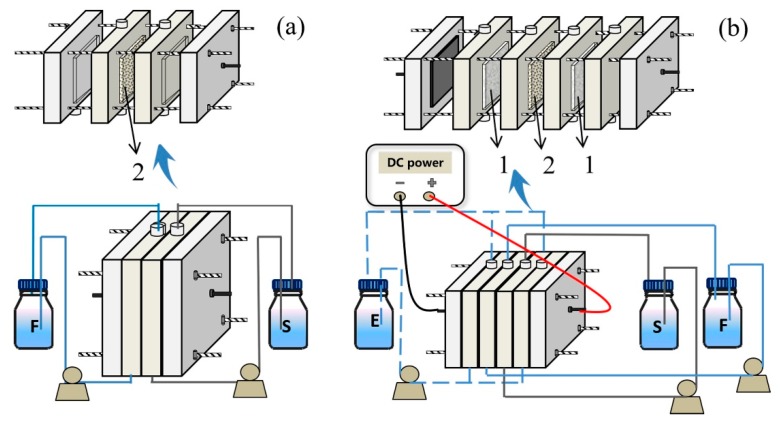
The experimental apparatus for the selective separation of VFAs (**a**) without the application of an electric field and (**b**) with the application of an electric field (1: Cation exchange membrane; 2: PIM; F: Feed solution; S: Stripping solution; E: Electrode solution).

Transport experiments were conducted at approximately 25 °C in a device that comprised two equal transport cells (thickness, 1 cm) in contact through a PIM membrane with an exposed membrane area of 20 cm^2^. The experimental apparatus is presented in detail in Figure 15a. The feed solution contained 250 mL of VFA (acetic and hexanoic acids) solution and the stripping solution contained 250 mL of Na_2_CO_3_ or HCl solution. The pH value in the feed solution was adjusted by the NaOH and HCl solution. The entire two cell solutions were driven by pumps at a certain flow rate of 300 mL min^−1^.

Unlike the above transport experiments, the ED experiment was performed using the ED stack comprised of two titanium electrode boards coated with rare metals (ruthenium) and four cells (Figure 15b). Four cells were separated by one prepared PIM and two CJMC-5 commercial cation exchange membranes. The thickness of the cells and the effective membrane area was also 1 cm and 20 cm^2^, respectively. The feed solution contained 250 mL 0.05 mol L^−1^ VFA (acetic and hexanoic acids) solution and the stripping solution contained 250 mL 0.1 mol L^−1^ HCl solution. Two electrode cells were pumped with the same concentration of 250 mL 0.1 mol L^−1^ H_2_SO_4_ solution. The constant current mode was supplied by a DC power supply (MCH-k305D, Shenzhen, China) with a current density of 5 mA cm^−2^. Other experimental conditions were the same as the above experiment.

In all experiments, samples for VFA (acetic and hexanoic acids) analysis were taken periodically from both cells using a micropipette and a chemical determination was performed using a high performance liquid chromatography (HPLC) unit equipped with a UV detector (210 nm) (Agilent Corp., Santa Clara, CA, USA). An ion exchange column (Aminex HPX-87H) (300 × 7.8 mm, Bio-Rad, Hercules, CA, USA) was used at a temperature of 60 °C with a 5 mmol L^−1^ H_2_SO_4_ solution as an eluent at a flow rate of 0.6 mL min^−1^.

### 3.4. Adsorption Capacity Test

The adsorption experiment was carried out using a CTA-60 wt% Aliquat 336 membrane to study its difference in the adsorption capacity of both acids. Membrane segments of an equal size were weighed and immersed into the feed solution containing 150 mL of 0.01 mol L^−1^ acetic and hexanoic acids, respectively. The solution was stirred using a constant temperature oscillator at 100 rpm and 1 mL samples were withdrawn at predetermined times, which were replaced with 1 mL of fresh aqueous feed solution for the purpose of keeping the aqueous feed solution volume constant. The samples were analyzed subsequently and the adsorption capacity of the membrane was obtained by calculation.

### 3.5. Data Processing

Adsorption capacity $q_{e}$ (mol g^−1^) of the membrane were calculated using Equation (3):

(3) $$q_{e}=\frac{\left(C_{i}-C_{e}\right)\cdot V}{m}$$

where $q_{e}$ is the equilibrium adsorption capacity for VFAs (mol g^−1^). $C_{i}$ and $C_{e}$ are the concentrations of the initial and equilibrium VFA feed solutions (mol L^−1^), respectively; V is the volume of the feed solution (L); and m is the measured mass of the PIM segments (g).

The extraction rate E (%) of the VFAs from the feed solution was calculated based on Equation (4):

(4) $$E=\frac{C_{0}-C_{f}}{C_{0}}\times 100\%$$

where $C_{0}$ is the concentration of VFAs in the feed solution after transport (mg L^−1^), and $C_{f}$ is the initial concentration of VFAs in the feed solution (mg L^−1^).

The recovery rate R (%) of VFAs from the PIM was determined by Equation (5):

(5) $$R=\frac{C_{S}}{C_{0}}\times 100\%$$

where $C_{s}$ is the concentration of VFAs in the stripping solution after transport.

The kinetics of the transport process through the PIM can be described by a first order reaction with respect to VFA concentration.

(6) $$\ln\left(\frac{C_{f}}{C_{0}}\right)=\mathrm{kt}$$

where k is the transport process rate constant (s^−1^), and t is the transport time (s).The relationship of $ln(C_{f}/C_{0})$ vs. time was linear, which was verified by the high values of the correlation coefficients (R^2^) ranging between 0.983 and 0.991.

The permeability coefficient P (µm s^−1^) was determined via Equation (7):

(7) $$P=\frac{V}{A}\times k$$

where V is the volume of the feed solution (m^3^), and A is the effective area of the membrane (m^2^).

The purity (%) of an individual VFA in the stripping solution was calculated using Equation (8):

(8) $$\mathrm{Purity}=\frac{C_{s}}{C_{t}}\times 100\%$$

where $C_{s}$ is the concentration of an individual VFA in the stripping solution after transport (mol L^−1^), and $C_{t}$ is the concentration of total VFAs in the stripping solution (mol L^−1^).

The average values of three parallel samples were utilized as the experimental data. The errors of all three experiments were all less than 5%. The obvious differences between each group was evaluated using Tukey’s multiple range tests, with *P* < 0.01 as an obvious difference.

## 4. Conclusions

The transport and recovery of VFAs (acetic and hexanoic acids) though a PIM containing Aliquat 336 and Cyphos IL 101 as the carrier were investigated. The more hydrophobic hexanoic acid was much more selectively transported than that of acetic acid through PIMs. PIM with 60 wt% Aliquat 336 as the carrier was proven to have a more efficient separation performance. A larger initial concentration of VFAs tended to give a much higher purity of hexanoic acid in the stripping solution, which was 89.3 % at the maximum concentration of 0.05 mol L^−1^. Furthermore, the use of Na_2_CO_3_ as a stripping solution achieved a much higher transport efficiency of the total VFAs when compared with HCl, whereas the purity of hexanoic acid in the stripping solution decreased from 81.8% to 50.8%. Additionally, the higher the concentration of HCl in the stripping solution, the lower the purity of the hexanoic acid. The efficient transport of the acid radical ions in an alkaline solution demonstrated that the transport mechanism was a coordinating mechanism of hydrogen bonding and ion-exchange. Moreover, the incorporation of electrodialysis and PIM resulted in a remarkable improvement in the transport efficiency of both acids, while selective separation was not achieved. The results in this study reveal that the PIM containing Aliquat 336 offers considerable potential for the selective separation of VFAs from aqueous solutions.

## Figures and Tables

**Table 1:** Permeability coefficient (P) and purity at different pH values.

pH	P (µm s^−1^)		Purity (%)
	Acetic Acid	Hexanoic Acid	
2	0.42	5.63	76.2
4	0.42	6.67	77.4
5	0.42	6.25	77.1
6	0.83	5.63	73.5
8	0.63	5.00	75.9
9	0.42	5.63	76.5
10	0.42	5.63	77.9

PIMs: PIM4; Feed solution: 0.01 mol L^−1^ acetic acid +0.01 mol L^−1^ hexanoic acid; Stripping solution: 0.1 mol L^−1^ HCl. Operation time: 12 h.

**Table 2:** The information of the PIM composition and proportion.

Membrane Number	Base Polymer (wt%)	Carrier (wt%)		Total Weight (g)	Thickness (µm)
	CTA	Aliquat 336	Cyphos IL101		
1	70	30		1.1	58.1 ± 5.1
2	60	40		1.1	57.8 ± 6.2
3	50	50		1.1	58.6 ± 5.6
4	40	60		1.1	60.4 ± 5.2
5	30	70		1.1	59.6 ± 8.7
6	40		60	1.1	61.4 ± 4.6
